# Supplementary figures and images for: Neurotoxicity of different amyloid beta subspecies in mice and their interaction with isoflurane anaesthesia
Source: PLoS One. 2020 Dec 3;15(12):e0242989. doi: 10.1371/journal.pone.0242989 (PMC7714346; doi:10.1371/journal.pone.0242989)

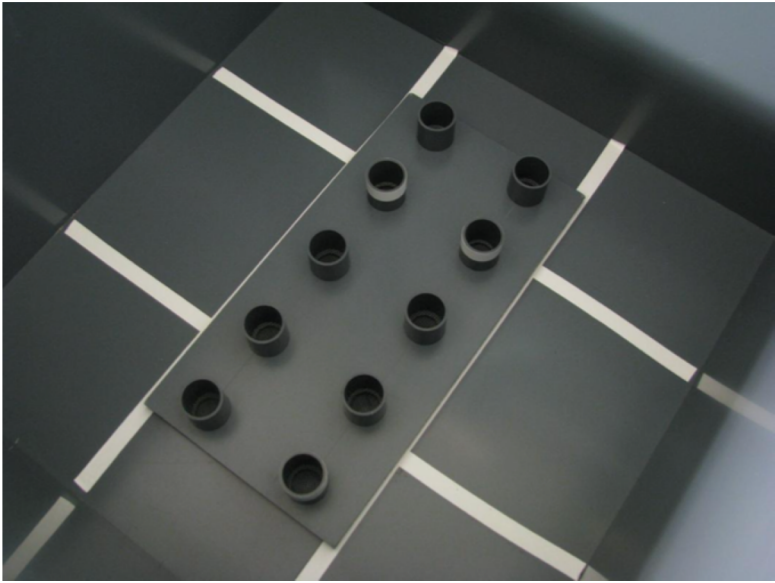

Supplement: S1 Fig — (PDF) [file pone.0242989.s001.pdf]
